# The public health implications of telematic technologies: An exploratory qualitative study in the UK

**DOI:** 10.1016/j.jth.2019.100795

**Published:** 2020-03

**Authors:** Judith Green, Andrey Romanovitch, Emma Garnett, Rebecca Steinbach, Daniel Lewis

**Affiliations:** a SUPHI, School of Population Health & Environmental Sciences, King's College London, UK; b Department of Social & Environmental Health Research, London School of Hygiene & Tropical Medicine, London, UK

**Keywords:** Telematics, Novice drivers, Collisions, Insurance, Determinants of health, Injury risk

## Abstract

**Introduction:**

Reducing motorised transport is crucial for achieving public health goals, but cars will continue to be essential for many in the medium term. The role of emerging technologies in mitigating the public health disadvantages of this private car use has been under-examined to date. Telematics are increasingly used by novice drivers in the UK to reduce insurance premiums. An exploratory study of novice drivers’ experiences of telematics identified implications for public health that warrant urgent further research.

**Methods:**

An exploratory qualitative study, using semi-structured interviews with 12 drivers aged 17–25 in three regions of the UK (Aberdeenshire, Hertfordshire and London).

**Results:**

Telematics were acceptable to young drivers, and reported to mitigate some negative health consequences of driving (injury risks, over-reliance on car transport), without reducing access to determinants of health such as employment or social life. However, there were suggestions that those at higher risk were less likely to adopt telematics.

**Conclusion:**

Market-based mechanisms such as telematics are potential alternatives to well-evaluated policy interventions such as Graduated Driver Licensing for reducing road injury risks for novice drivers, with a different mix of risks and benefits. However, claims to date from insurance companies about the contribution of telematics to public health outcomes should be evaluated carefully to account for biases in uptake.

## 1 Introduction

The public health disadvantages of transport systems dominated by private car use are well documented, and reducing reliance on motorised transport is crucial for sustainability and health. However, in the medium term, cars will remain essential for many, particularly in rural areas. There is therefore a need to identify public health interventions which can mitigate negative consequences such as road traffic injury, pollution and inequalities arising from transport exclusion. The burden of injury is disproportionately borne by young novice drivers, who are over-represented in road crashes (Williams, 2003; Cassarino and Murphy, 2018). To date, Graduated Driver Licensing (GDL) policies have been identified as effective in reducing this burden (Williams, 2017; Zhu et al., 2016), with considerable uptake across Australasia and north America, but less in Europe (Christie et al., 2017), in part because of concerns around the potential impact of driver restrictions on other determinants of health, such as access to employment and social life. A study of young drivers in the UK (Steinbach et al., 2016; Christie et al., 2017; Green et al., 2018), conducted in preparation for the planned implementation of a GDL programme in Northern Ireland, mapped two key pathways through which GDL might impact on public health: through reducing road injury (a positive impact), and through changing transport access and mode choices in ways that might (in the short term) increase transport exclusion (a negative impact) (Steinbach et al., 2016). This study also noted the potential of telematics (sometimes called intelligent vehicle technologies, or more colloquially, the ‘black box’) to achieve road injury gains with fewer costs in terms of transport equity, particularly in rural areas. In summary, young drivers believed they would be rewarded for their ‘good’ driving by lower premiums and parents welcomed the option to ‘delegate’ policing of their adult children's driving to technology, although there were some concerns from those in Northern Ireland about the potential for surveillance. However, at the time of the study (2015), few had any direct experience of using telematics.

Smart car technologies have developed rapidly since that study (Tselentis et al., 2017). Telematics technologies utilise Global Positioning Systems to map journeys in real time together with a variety of in-car diagnostic sensors (e.g. accelerometers) to derive information about driving style (speed, braking times, cornering), electronically sending these data to insurance companies and (sometimes) to the driver's smart phone. In the UK, telematics are primarily marketed to young drivers as a “clever way of using technology to get an insurance quote that reflects the way you drive” (Comparethemarket.com 2019): a way of replacing aggregate risk assessments with personal ones and thus making insurance more affordable.

Telematics, as promoted through insurance markets, potentially reduce several negative outcomes of car use (Tselentis et al., 2017). In principle, they reduce road crash risks by obliging or encouraging young drivers to consent to direct monitoring (and on occasion curtailing) of risky driving behaviours such as driving at night or on routes that are high risk for road crashes (Ayuso et al., 2014; Verbelen et al., 2018). They encourage lower mileage and fuel-efficient driving styles, and thus, at least at the margins, reduce pollution (Manzie et al., 2007; Tselentis et al., 2017). Finally, they can facilitate car sharing platforms, which might reduce private car ownership and usage (Cho et al., 2006). If widely adopted, telematics therefore offer an alternative to legislative initiatives such as GDL. Mechanisms used to influence driver behaviour include: financial incentives through reduced premiums; ‘gamification’ such as motivational feedback to the user's smartphone, points, ‘badge’ rewards, positive comments about driving style; and warnings, reduced scores or even removal of cover for disfavoured driving routes or practices. The UK insurance industry claims that uptake of these technologies over the last three years has reduced casualty rates among young drivers (BIBA, 2018). However, to date, there is little research on how and why telematics have been taken up. Given recent increases in uptake, their potential for impact on public health, and the claims made by insurance companies, evidence on how these technologies are used in practice is vital. To provide initial insight into how and why the key target users select (or not) telematics products, and what the implications for public health might be, we undertook a small scale exploratory study with young drivers.

## 2 Material and methods

This exploratory study recruited a purposive volunteer sample of drivers aged between 17 and 25, to include a range of rural and urban settings and driving experiences. Recruitment was via driving instructors and a large employer (asking contacts to pass on invitations to take part) in three regions of the UK; Hertfordshire, Aberdeenshire and London. Semi-structured interviews included prompts on: driving histories and current use; knowledge of telematics technologies; experiences of using telematics (for self and peers); how decisions about telematics were made; concerns about data monitoring; and experiences of other in-car technologies. Interviews were audio-recorded, and anonymised transcripts analysed using qualitative thematic content analysis. Initial coding identified key themes, and analysis of these was focused deductively at identifying rationales for accepting or declining telematics, and at accounts of how telematics technologies affected their own, and known others’, driving. Sampling continued until saturation: i.e. the point where no new themes were identified from coding transcripts. The final sample of 12 (five male, seven female) had held licences for one year or less (n = 5), between 1 and 3 years (n = 4), or over 3 years (n = 3). Six were in employment, five were full time students, and one was an apprentice. Approval was granted by Ethics Committees at KCL (MR/17/18–248) and LSHTM (15103); all participants (I1 – I12 in extracts below) provided informed consent.

## 3 Results

### 3.1 Choosing telematics

All participants had considered telematics, and were familiar with the technology; six had chosen telematics for at least one policy. Cost saving was the main incentive for accepting (“it all came down to price really” (I2); “without the black box, it would have been £2500 – I could only get the £1000 price with it” (I11)), with some reporting that parents paid for insurance and had selected a telematics-based insurance product. Those who declined (ever, or for their current policy) reported reasons such as: curfew constraints (I1, I9); speed limitations (I3); the savings accrued from good driving would not take effect on premiums until the following year (I3); financial gains only being worthwhile in the first year of driving (I11). Only one participant reported concerns about monitoring (I10), related to parental concern about the additional stress for a new driver from constant feedback.

Few participants had any detailed knowledge of, or interest in, how the technology worked. None reported concerns about data privacy or data ownership, although one ex-user noted (on reflection) that it was “weird” (I1) that the box remained installed in their car, despite no longer being with the installer's insurer.

### 3.2 Reported impact on driving behaviour

Once telematics had been adopted, the rewards systems (such as colour coded awards) were reported as acceptable and effective by most: “if you brake too hard it will come up with a flag on the map – I kind of like getting a good score” (I7); “I did try and get a good a score as I could” (I6); “it does help … a good indication if you weren't driving so good, to readjust your driving style” ((I2). Some felt that it was particularly useful for them, as novice drivers, to have direct feedback on their driving “to get you into the habit of driving safely at the start” (I8).

Those who reported being unaffected by feedback attributed this either: to the fact that savings would not be applied until the following year, so monitoring did not directly affect premiums, rather than it not have the capacity to do so (“[it was] pointless [as] I was going to shop around … it did not really incentivise me to drive really, really sensibly” (I1)); or to their well-established safe driving styles, which pre-existed installation of a black box (“I don't speed, I don't care about accelerating fast, I don't brake harshly – that was how I was driving anyway” (I4)).

Although no participant admitted to breaking road rules or driving poorly themselves, some had declined telematics because of potential limitations: “I didn't want the monitoring, on the speed” (I10). Several cited peers who had either rejected telematics or had their insurance cancelled because of risk taking: “one stayed past 12 and got a phone call … one got his insurance cancelled going over 100 miles an hour” (I1); “one friend took it off as it was too annoying … I think he was a fast driver, and he did not like the constraints” (I3). Others reported deliberately driving on roads that were not covered by the black box (in more rural areas) so that they could “enjoy the windy roads without getting picked up” (I5).

### 3.3 Impact on broader determinants of health

Access to employment, goods and services and social networks are essential determinants of health. Meeting these requires access to a private car for many in settings with limited public transport (Christie et al., 2017). Telematics products which limit mileage or routes might in theory curtail this access, but there was no evidence that current provisions did so in practice. Those requiring or desiring high mileage cited this as a reason for choosing higher premiums rather than telematics: “the restrictions do not justify the £100 saving, [you couldn't] stay out between 1 and 4am” (I1). Some users did report holding policies with incentives to reduce mileage or avoid routes with high crash risks. However, they reported that journeys that had to be made (such as to work in the rush hour) were still undertaken, despite incurring ‘poor scores’. For more discretionary journeys, changes were made which reduced either mileage or risk exposure. These could entail healthier mode choice: “if I can avoid driving in town, I will, as I know it will put the score down, so I will walk instead” (I7).

## 4 Discussion

This small study was designed to be explorative: it is not known how far the views here represent those of 17–25 year olds more generally. However, the findings suggest that telematic technologies have potential to improve the public health. Telematics offered as an incentive to reduce insurance premiums were broadly acceptable to young drivers, in contrast to earlier reports of drivers’ concerns about surveillance, at least from some parts of the UK (Christie et al., 2017). Users reported that telematics encourage safer driving behaviour, which may help reduce miles driven, and morbidity and mortality from road collisions involving novice drivers. As telematics were not adopted by those for whom the restrictions would be burdensome, these reductions may take place without increasing transport exclusion among young people in areas less well served by public transport. As an intervention, telematics therefore have, in principle, potential advantages over initiatives such as GDL, which require legislation, statutory changes across a range of domains (licensing, driver education, policing) and enforcement. As a market mechanism, telematics require no such publicly-funded infrastructure, although insurance companies are requesting tax breaks on technologies due to the claimed public health advantages.

However, our findings suggest that further research is urgently needed on whether these potential benefits are realised in practice, for three reasons. Most importantly, although many of those with devices self-reported a positive impact on their driving styles, some chose not to install them to avoid curfews or mileage constraints. Others reported that peers undertaking ‘risky’ driving (late at night, speeding, enjoying windy roads) chose not to use telematics. This raises the possibility of insurance company claims about effectiveness being biased by selective uptake by those least likely to be at higher risk. Unlike GDL schemes (which would be universal), the benefits of telematics currently rely on users opting in. Second, it is not known how far any positive impacts of black box feedback in the early years of driving are sustained over subsequent years. More behavioural research on the longer term effectiveness of mechanisms such as smart-phone feedback and financial incentives is needed to assess the likely effectiveness of these market-based interventions as a way of reducing risks for young drivers. Third, in-car technologies are developing rapidly. Young drivers in this study reported additional smart car technologies which directly assisted safer braking, cornering and manoeuvring: these technologies may make indirect behavioural change techniques less relevant in the near future. Further research on the potential for smart car technologies to mitigate the negative health consequences of driving should engage with how these technologies are used in practice, identify who is and isn't adopting them, and evaluate what impacts they are having on road traffic crashes involving novice drivers.

## 5 Conclusions

Novice drivers who use telematics technologies in the UK reported that these reduced their risky driving, with few other negative impacts on determinants of public health. However, there are suggestions that uptake is selective, unlike for GDL schemes, which would have a universal impact. Research is urgently needed on whether telematics do offer as effective a way to reduce road injury risks for young drivers as (more costly) interventions such as GDL, and also on what broader impacts in-car digital technologies and insurance market mechanisms have on driving practices and the wider determinants of public health.

## Author statement

**Judith Green**: funding acquisition, conceptualisation, methodology, analysis, writing- original draft. **Rebecca Steinbach**: funding acquisition, conceptualisation, supervision, writing-review & editing. **Daniel Lewis**: funding acquisition, conceptualisation, supervision, writing-editing and reviewing. **Emma Garnett**: funding acquisition, conceptualisation, analysis, writing-review & editing. **Andrey Romanovitch**: investigation, analysis, writing-review & editing.

## Funding

This study was funded by the LSHTM-King's Research Collaboration, funded through the 10.13039/100004440Wellcome ISSF grant 204823/Z/16/Z.
